# Cost-effectiveness of recombinant influenza vaccine compared with standard dose influenza vaccine in adults 18–64 years of age

**DOI:** 10.1016/j.vaccine.2024.07.008

**Published:** 2024-07-05

**Authors:** Mary Patricia Nowalk, Kenneth J. Smith, Jonathan M. Raviotta, Angela Wateska, Richard K. Zimmerman

**Affiliations:** a University of Pittsburgh School of Medicine, Department of Family Medicine, Pittsburgh PA, 15261 USA; b University of Pittsburgh School of Medicine, Department of Medicine, Pittsburgh PA, 15261 USA

**Keywords:** Influenza, Hospitalization, Cost-effectiveness, Recombinant influenza vaccine, Standard dose influenza vaccine

## Abstract

**Background:**

The Advisory Committee on Immunization Practices (ACIP) uses the Evidence to Recommendations Framework that includes cost-effectiveness analyses (CEA) for determining vaccine recommendations. ACIP’s preference for protecting adults ≥ 65 years is enhanced vaccines, including recombinant influenza vaccine (RIV4), adjuvanted or high dose influenza vaccine. Less is known about the CEA of enhanced vaccines for younger adults.

**Methods:**

We used decision analysis modeling from a societal perspective to determine the cost-effectiveness, measured in quality adjusted life years (QALYs), of RIV4 compared with standard dose quadrivalent influenza vaccine (SD-IIV4) in adults 18–64 years old. Model inputs included 2018–2020 vaccine effectiveness (VE) estimates based on medical record data from a large local health system, 2019–2020 national vaccination and influenza epidemic parameters, with costs and population distributions fitted to the season.

**Results:**

Among adults ages 18–64 years, RIV4 cost $94,186/QALY gained, compared to SD-IIV4. Among those 50–64 years old, RIV4 was relatively more cost-effective ($61,329/QALY gained). Cost-effectiveness estimates for 18–64-year-olds were sensitive to the absolute difference in VE between SD-IIV4 and RIV4, among other parameters. Use of RIV4 in 18–64-year-olds would result in fewer cases (669,984), outpatient visits (261,293), hospitalizations (20,046) and deaths (1,018) annually. The majority (59 %; 597 of 1018) of the decreases in deaths occurred in the 50–64-year-olds.

**Conclusions:**

While RIV4 was effective and cost-effective relative to SD-IIV4 for both 50–64-year-old and 18–64-year-old adults, cost-effectiveness was sensitive to small changes in parameters among 18–64-year-olds. Because substantial public health benefits occur with enhanced vaccines, health systems and policy makers may opt for preferential product use in select age/risk groups (e.g., 50–64 year olds) to maximize their cost-benefit ratios.

## 1. Introduction

As influenza continues to cause significant morbidity and mortality across the U.S. and globally, new vaccine formulations are being brought to market in an effort to improve vaccine effectiveness (VE). New technologies such as cell-cultured (ccIIV), recombinant (RIV) and adjuvanted influenza (Adj-IV) vaccines, as well as high-dose influenza vaccine (HD-IIV) have shown promise as more effective means of attenuating annual influenza epidemics, than older, standard dose vaccines (SD-IIV) that rely on growth in eggs, whose adaptations may render them less potent against the virus. A randomized controlled trial reported significant relative vaccine efficacy of RIV4 of 30 % among adults ≥ 50 years old against RT-PCR confirmed influenza-like illness [1]. A retrospective test-negative case-control study comparing VE of RIV4 and SD-IIV4 among outpatients found significant VE of both vaccines, but a non-significant, 11 percentage point difference in relative VE (rVE) between the two vaccine types [2]. One retrospective cohort study of adults ≥ 65 years reported significant rVE of RIV4 compared with SD-IIV4 against hospital encounters, [3] another found RIV4 to be more effective than SD-IIV4 for preventing influenza hospitalization in all adults ≥ 18 years old and adults 18–64 years [4].

Development of vaccines, followed by testing for safety and effectiveness is an expensive undertaking, typically resulting in high costs for new products relative to products that are based on older, more time-tested technologies. Thus, in addition to vaccine effectiveness trials, cost-effectiveness analyses (CEA) that account for costs of vaccine and illness, rVE and other factors such as uptake and acceptability due to route of administration (needle vs. nasal spray), etc., may be performed. Comparative CEA of two or more vaccine products provide valuable insight for both vaccine policy makers and vaccine purchasing decision makers. The Advisory Committee on Immunization Practices’ (ACIP) latest influenza recommendations include a preference for high dose, recombinant or adjuvanted influenza vaccines for individuals aged 65 and older due to their higher prevalence of high-risk medical conditions and the higher likelihood of serious influenza complications [5]. However, prevalence of medical conditions that increase the risk of complications from influenza is on the rise among younger adults. Therefore, a CEA comparing enhanced vaccines and standard influenza vaccines may provide a basis for lowering the age for preferential recommendation of enhanced influenza vaccines.

This study was designed to estimate the cost-effectiveness of RIV4 compared with SD-IIV4 in adults ages 18–64 years and 50–64 years old from the societal perspective, separately examining potential policy options that decrease ages for preferred recombinant influenza vaccine use to either 18 or 50 years, respectively. Base case VE data from two studies [2,4] that used electronic medical record (EMR) data from a large health system provided the basis for VE estimates used in the models. This health system purchased primarily RIV4 for use in its physician’s offices and other healthcare facilities.

## 2. Methods

Using a decision tree model, the cost-effectiveness and expected public health outcomes (cases, outpatient visits, hospitalizations, and deaths prevented) of influenza vaccination using the RIV4 (Flublok^®^) compared to SD-IIV4 were estimated for adults aged 18–64 years. In the primary analysis, two strategies were compared: vaccination with SD-IIV4 vs RIV4 among adults 18–64 years old: 1) under base case conditions, and 2) in sensitivity analyses.

### 2.1. Model description

**Fig. 1.**
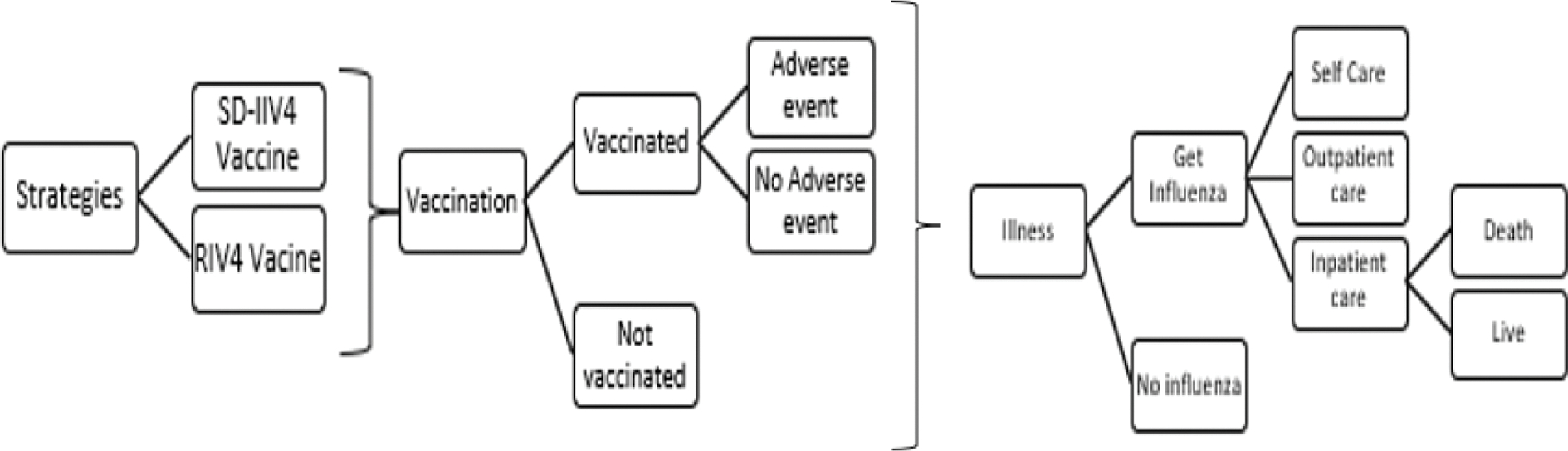
Model diagram.

The base case model is presented in Fig. 1. Vaccination status was assigned using the CDC estimates referenced in Table 1. Adverse events were assessed using clinical trial data, and the likelihood of infection was determined from the national influenza attack rate. Each of four infection conditions: self-care, outpatient care, inpatient care or death was assigned costs and lost QALYs. The cost of mortality included the costs of life-saving care. The self-care condition included the cost of over-the-counter analgesics for treatment of fever and pain. Medically attended cases were assigned relevant treatment costs and QALYs based on hospitalization status and the probability of being a high-risk patient.

Three outcomes from influenza infection were modeled: recovery, hospitalization with recovery, and death. The model excluded all other causes of death, assuming that vaccination strategy does not affect mortality from other causes. This assumption may bias against vaccination.

### 2.2. Parameters

Model parameters are shown in Table 1. When possible, costs, utilities, and probabilities were selected from the most current and robust data sources, as noted. Costs were adjusted to 2020 US dollars using the US Consumer Price Index. Likewise, population distributions and risk stratification were adjusted to 2020 values using weights derived from 2019 U.S. Census data [6]. The 2019–2020 national influenza vaccination and influenza attack rates provided the probabilities of vaccination and of an unvaccinated individual becoming ill, respectively. The probability of illness for vaccinees was derived from primary data collected from the EMR as the product of the annual attack rate and the age- and risk-adjusted vaccine effectiveness (VE). Base-case vaccine effectiveness values were derived from a retrospective test negative case control study using data from the same health system EMR [2,4]. VE estimates against all non-hospitalized influenza were assumed to be the same as VE against outpatient influenza illness and VE values for the 50–64-year age group were assumed to be the same as those for the entire 18–64-year group. High-risk determinations were made based on evidence of medical conditions described by Molinari et al. [7] and Carias et al. [8] Probabilities of high-risk conditions are from Molinari et al. [7] and costs associated with flu in persons without/with high-risk conditions are from Carias et al. [8] Absolute increase in VE was computed by subtracting the VE of SD-IIV4 from the VE for RIV4.

QALYs were used to measure duration and quality of life in cost-effectiveness comparisons. The analysis took a societal perspective where costs included the costs of vaccination, adverse events, illness, and lost productivity. Two age groups of the US population were analyzed; adults aged 18–64 years and a sub-analysis of adults aged 50–64 years.

Cost-effectiveness of each vaccine was compared by generating incremental cost-effectiveness ratios (ICER) in each age group. ICER = the difference in cost between the two vaccine strategies divided by the increase in effectiveness of one strategy over the other. The ICER outcome equals the cost per QALY gained. A willingness-to-pay threshold of ≤$100,000/QALY gained was used as an indicator of cost-effectiveness.

Public health results were derived from the model-based proportion of the cohort encountering these influenza illness health states multiplied by the 2020 US population size for persons ages 18–64 years (199,841,852) [9] and for persons ages 50–64 years (62,788,435) [9].

All parameters were analyzed in one-way sensitivity analyses across literature-based value ranges to validate the model’s function and to identify parameters whose variation changed the favorability of strategies. Two-way sensitivity analyses were generated to examine the effect of varying the cost of RIV4 across a range of literature-based VE values. The model did not account for possible indirect effects (herd immunity).

## 3. Results

Table 2 displays the results of the primary analysis including the per-person total cost of the vaccine strategies, the difference in cost between strategies, the effectiveness of each strategy, the difference in effectiveness between the two strategies and the cost per QALY gained or ICER. In the base case analysis for adults ages 18–64 years, vaccination with RIV4 cost $94,186/QALY gained compared to SD-IIV4. When the analysis was restricted to adults aged 50 to 64 years, the difference in cost was lower and the increase in effectiveness was greater resulting in an ICER of $61,329/QALY gained, thereby increasing RIV4 favorability.

One-way sensitivity analyses were examined for both age groups using the base case parameters plotting the decrease in cost/QALY gained against increasingly large differences in VE between the two vaccines. We separately show effectiveness in preventing outpatient and inpatient influenza to depict each of their effects on results, given much greater likelihood of outpatient vs. inpatient influenza and much higher costs associated with inpatient stays vs. outpatient treatment. As shown in Fig. 2a, which considers preventing outpatient influenza in adults 18–64 years of age, RIV4 costs more than the $100,000/QALY gained willingness-to-pay threshold if the absolute difference in VE between SD-IIV4 and RIV4 is approximately 4 % or less; whereas, for 50–64-yearolds, RIV4 costs less than $100,000/QALY gained throughout the examined range. When considering effectiveness in preventing inpatient influenza in either age group (Fig. 2b), RIV4 costs more than $100,000/QALY gained when the difference in VE between vaccines is less than 19 % in 18–64-year-olds or less than 13 % in 50–64-year-olds.

Two parameters whose joint variation might have substantial effects, i.e., RIV4 cost and the absolute increase in RIV4 VE over SD-IIV4 VE, were simultaneously varied in two-way sensitivity analyses. In these scenarios, the vaccine cost is the Centers for Medicare and Medicaid Services’ payment allowance for an adult dose of FluBlok^®^, CPT 90682, in the 2019–2020 influenza season ($56.06) and VE is represented as the *difference* in effectiveness between SD-IIV4 and RIV4. For example, if SD-IIV4 were 30 % effective and RIV4 were 40 % effective, the plotted value is the difference between those VE values, or 10 %. Fig 3a. and Fig 3b. depict the areas where each vaccine is favored when RIV4 costs and differences in VE for outpatient influenza prevention were varied in adults 50–64 years old (3a) and 18–64 years old (3b). The base case overall outpatient VE difference between the vaccines is 5 %, marked by a vertical line. When considering VE for preventing inpatient influenza in 50–64-year-olds, a similar picture emerges (Fig. 4a and Fig 4b.), but with greater potential for RIV4 becoming unfavorable if baseline VE differences of 20 % are not maintained in 50–64-year-olds.

To confirm these findings, Table 3 depicts those parameters whose individual variation over ranges listed in Table 1 resulted in RIV4 becoming unfavorable at a $100,000/QALY threshold (all remaining parameters were similarly varied but did not affect RIV4 favorability). In 50–64-year-olds, variation of outpatient SD-IIV4 VE, hospitalization VE for either vaccine, or influenza attack rates could result in RIV4 becoming unfavorable. In 18–64-year-olds, variation of several additional parameters could affect RIV4 favorability, including cost of RIV4 and RIV4 VE against outpatient illness. Relatively small changes to base case values could cause RIV4 to cost more than $100,000/QALY gained. For example, RIV4 would no longer be favorable at this threshold in either age group if RIV4 VE in preventing hospitalization were < 39.9 % or if influenza attack rates were < 10.2 %.

Potential public health effects on populations vaccinated with either SD-IIV4 or RIV4 are presented in Table 4. In the 18–64-year-old population (N = 199,841,852), the RIV4 strategy would offer better public health protection than the SD-IIV4 strategy resulting in 669,984 fewer influenza cases, 261,293 fewer outpatient encounters, 20,046 fewer hospitalizations and 1,018 fewer deaths per year in the U.S. Similarly, in the 50–64-year-old population, use of RIV4 instead of SD-IIV4 would result in 210,502 fewer influenza cases, 90,516 fewer outpatient encounters, 8,926 fewer hospitalizations and 597 fewer deaths per year in the U.S. The majority (59 %; 597 of 1018) of the decreases in deaths occurred in the 50–64-year-olds. Importantly, model results assume that decreases in hospitalization and mortality are directly proportionate. Thus, in base case analyses, RIV4 use resulted in 11 % decreases in both hospitalization (e.g., 8,926/80,730 for 50–64-year-olds) and mortality (597/5,407 for 50–64-year-olds). Relaxing this assumption and allowing influenza mortality to vary independently from hospitalization results in RIV4 becoming unfavorable at a $100,000/QALY threshold if influenza mortality with RIV4 use decreases less than 6.4 % in 50–64-year-olds or less than 11 % in 18–64-year-olds (data not shown).

## 4. Discussion

Using prices published in CMS’s vaccine price list, real-world data derived from a single multi-hospital system and a retrospective test-negative case-control design, we found that among working age adults 18–64 years old, RIV4 was more effective, and more cost-effective than SD-IIV4 when using a $100,000/QALY gained willingness-to-pay threshold and combining both outpatient and inpatient outcomes. Among the oldest of these adults (50–64 years), RIV4 had a better cost-effectiveness profile, with a lower cost per QALY gained than among the entire group that included younger adults 18–49 years of age. These findings may be explained by lower rates of preexisting high-risk conditions in younger adults compared with the 50–64-year-old subgroup for whom the ICER was significantly below the $100,000/QALY gained willingness-to-pay threshold. The CEA is sensitive to assumptions in the 18–64-year-old group. We also found that use of an enhanced vaccine such as RIV4 has important public health benefits by preventing more deaths, hospitalizations, outpatient visits and influenza cases.

Interpretation of these data should be approached with caution because they were not robust in sensitivity analyses for 18–64-year-olds. Inpatient RIV4 VE, inpatient and outpatient SD-IIV4 VE, influenza attack rates, and the cost of RIV4 had the largest effects on the ICER among 18–64-year-olds in this study. Moreover, small variation in the base case parameters, especially rVE of the two vaccines against influenza-related hospitalization and the influenza attack rate changed the cost-effectiveness ratios for RIV4.

The factor(s) exerting the largest effect on ICERs, especially of RIV4, vary from study to study. In a study of influenza vaccines in general, the variables affecting ICERs differed by age/risk group. Among non-high risk 18–64-year-olds, the ICERs were most sensitive to changes in VE and the probability of influenza illness; whereas, in high-risk groups, the cost of illness-related medical care and the probability of influenza illness were the most influential factors [10]. In a comparison of ccIIV4 and RIV4 in 18–64-year-olds, the driving variable for cost-effectiveness was VE of ccIIV4 [11].

Much of the influenza vaccine cost-effectiveness research has focused on adults ≥ 65 years of age and comparisons between HD-IIV and SD-IIV, [12,13] and between Adj-IV and SD-IIV, [14,15] finding the newer formulations to be cost-effective compared with SD-IIV. In its evidence-to-recommendations framework, the CDC’s cost-effectiveness analysis reported variability in ICERs of high dose and adjuvanted vaccines based on VE estimates and season severity, with 20 % of scenarios being cost saving among adults ≥ 65 years of age [16].

In contrast, there has been less research comparing cost-effectiveness of influenza vaccines among adults younger than age 65. Uruena et al. reported better cost-effectiveness of ccIIV4 over SD-IIV4 among adults < 65 years old in Argentina; [17] Raviotta et al. reported potential economic benefit of HD-IIV and RIV4 among high risk 50–64-year-olds in the U.S., [18] and Maschio et al. compared cost-effectiveness of ccIIV4 and RIV4 among adults < 65 years old in the United Kingdom, [11] reporting that RIV4 was not more cost-effective than ccIIV4. DeLuca et al. modeled cost-effectiveness of influenza vaccination across age and risk groups and found that influenza vaccination in general was cost-effective at a $100,000/QALY gained willingness-to-pay threshold for all age/risk groups except for non-high-risk younger adults 18–49 years of age [10]. These findings align with ours that suggest that inclusion of younger adults in the analyses reduced favorability of RIV4.

### 4.1. Strengths and limitations

The strengths of this study include use of real-world data and two-way sensitivity analyses that allowed for a range of costs and of outpatient VE to be used. All models were estimates of reality but may have missed important variation; thus, we varied parameters widely in sensitivity analyses.

Our model used primary data collected from the EMR of a large health system to calculate vaccine effectiveness for the stratified groups. The observed results may not reflect other populations if they differ significantly from the local sample. Vaccine uptake was assumed to be identical for both strategies and all age groups as RIV4 was purchased by this health system and was widely available. The actual uptake rates may differ from the observed values in other health systems where patients have other vaccine options. VE values for the 50–64-year age group were assumed to be the same as those for the entire 18–64-year group, it is possible that VE was lower for the older group which could result in a higher cost-effectiveness ratio.

Additionally, future influenza epidemics may differ from the 2018–2019 and 2019–2020 seasons. The 2018–19 influenza season was considered to be long and moderately severe, with two influenza A waves, first A(H1N1) followed by A(H3N2), [19] and 2019–20 was somewhat milder with a B wave followed by an A(H1N1) wave [20]. We chose these two seasons because we based the VE estimates on two publications that made VE estimates for those two seasons. Except for 2019–2020, the next several years saw a disruption of typical influenza seasonality due to the COVID-19 pandemic. Our data indicate that relatively small changes in RIV4 VE against hospitalization, or influenza attack rates would reduce the favorability of RIV4 at a $100,000/QALY willingness-to-pay threshold. In a different season with circulation of more or less virulent strains, or one with a worse match between circulating and vaccine virus strains, VE may differ and the CEA would need to be updated with new rVE values.

## 5. Conclusions

Over the decades since vaccination of all eligible individuals 6 months of age and older has been recommended in the U.S., cost-effectiveness analyses have provided evidence that vaccination is generally a cost-effective public health intervention to reduce the burden of influenza in the population. Modest vaccine effectiveness of standard dose egg-based influenza vaccines has led to the development of new vaccine technologies including HD-IIV, Adj-IV, ccIIV, and RIV. But these new technologies come at a relatively high cost. Effective vaccine policy is that which protects those most at risk and efficiently uses healthcare resources. Thus, policy makers must balance the costs of new vaccines with improvements in effectiveness and reduction of disease burden specific to different age and risk groups.

This study showed that RIV4 is cost-effective relative to SD-IIV4 for adults 50–64 years. Use of RIV4 instead of SD-IIV4 among all working-age adults 18–64 years would result in thousands fewer influenza-related hospitalizations, office visits and influenza infections. However, RIV4′s cost-effectiveness relative to SD-IIV4 is sensitive to assumptions including the proportion of the group with high-risk conditions and characteristics of the influenza season that can affect cost or effectiveness and result in an unfavorable cost-effectiveness ratio. Health systems and policy makers may opt for preferential product use in select age/risk groups (e.g., 50–64 year olds) to maximize their cost-benefit ratios.

## Figures and Tables

**Fig. 2a.**
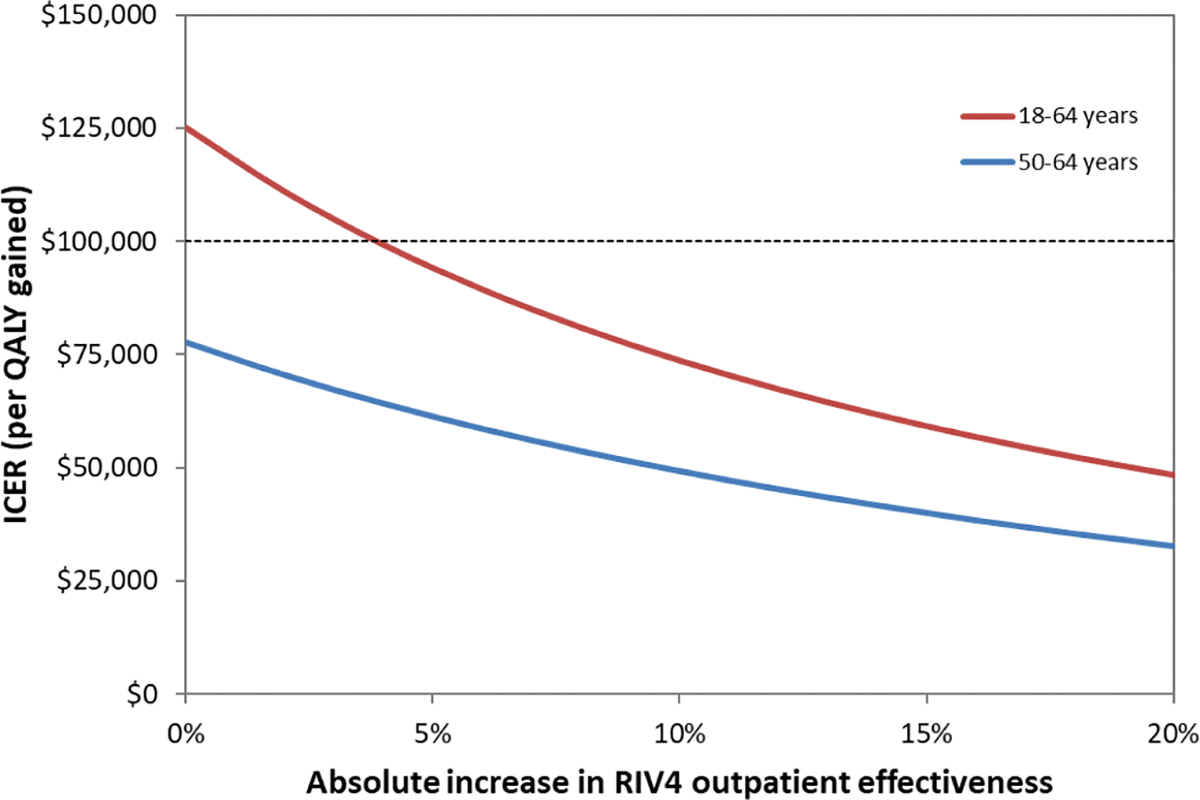
Sensitivity analysis showing the decrease in cost per QALY gained plotted against the increase in the difference in effectiveness between RIV4 and SD-IIV4 in *outpatients* 18–64 years and 50–64 years.

**Fig. 2b.**
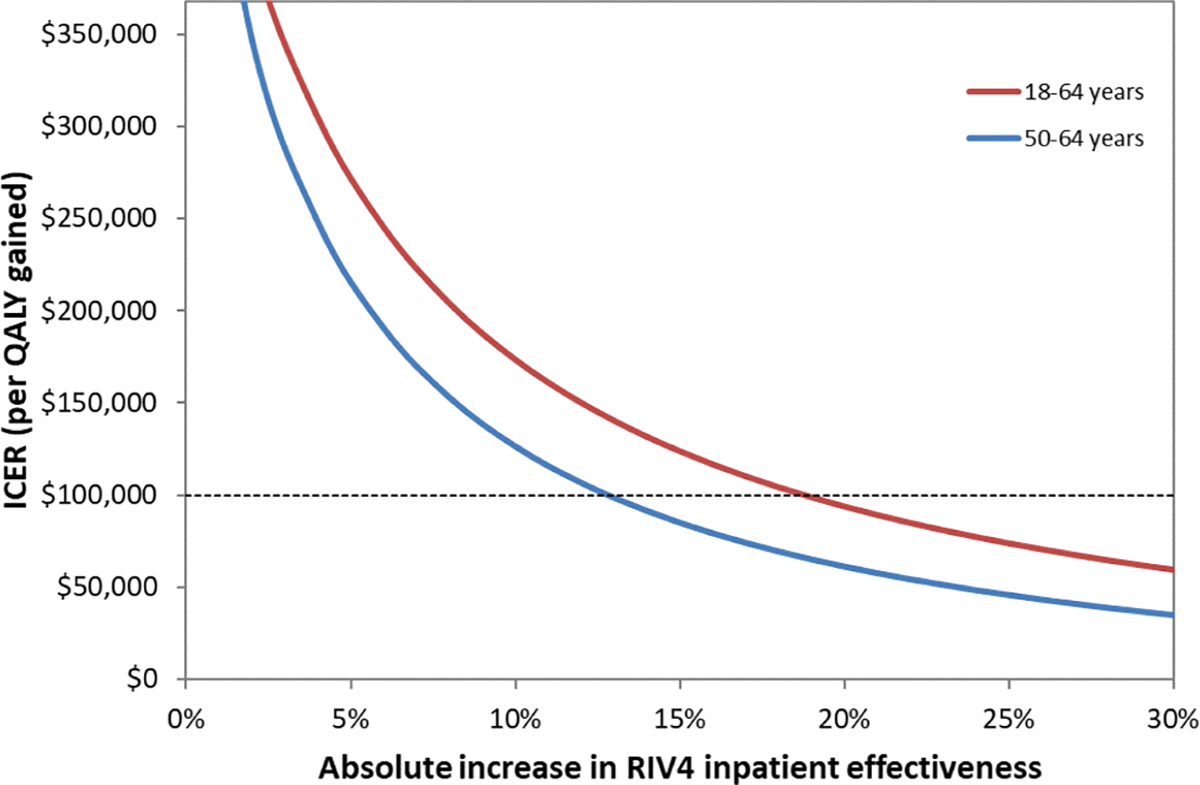
Sensitivity analysis showing the decrease in cost per QALY gained plotted against the increase in the difference in effectiveness between RIV4 and SD-IIV4 in *inpatients* 18–64 years and 50–64 years.

**Fig. 3a.**
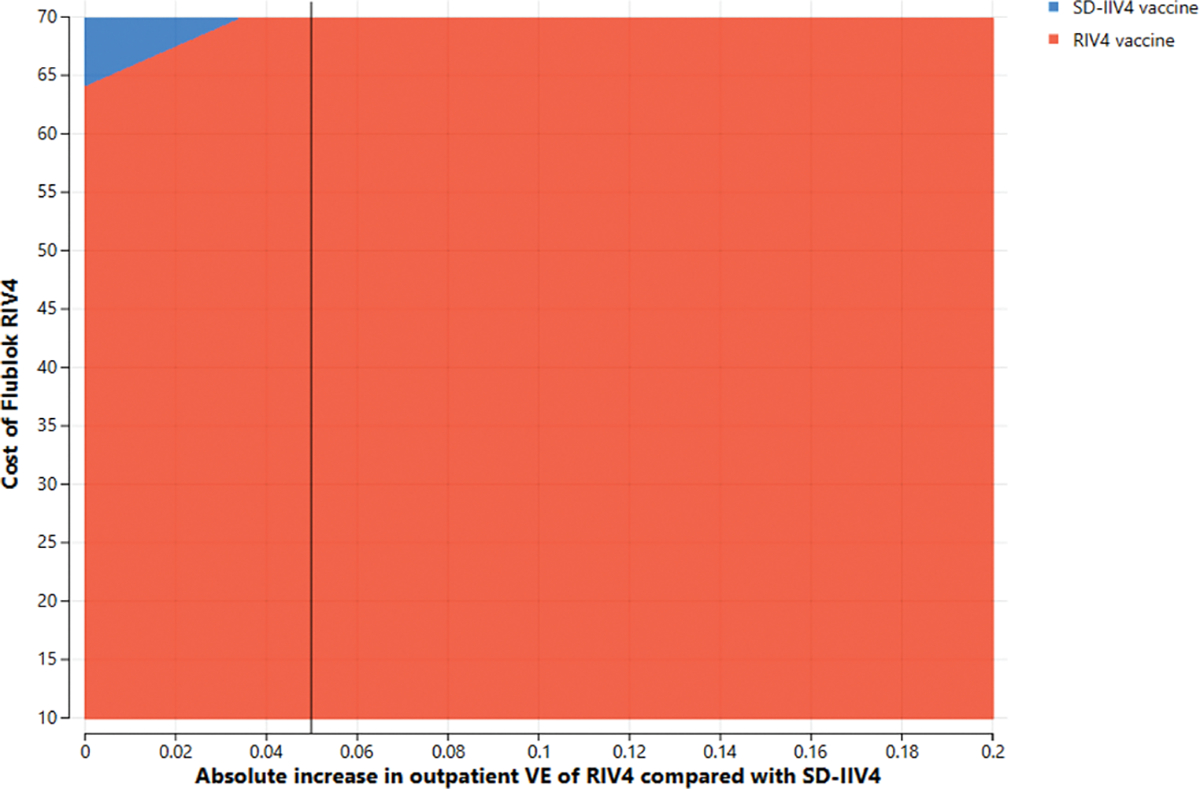
Two-way sensitivity analysis of absolute difference in *outpatien*t vaccine effectiveness between RIV4 and SD-IIV4 by cost of a dose of RIV4 in 50–64-year-olds at a $100,000/QALY gained willingness-to-pay threshold.

**Fig. 3b.**
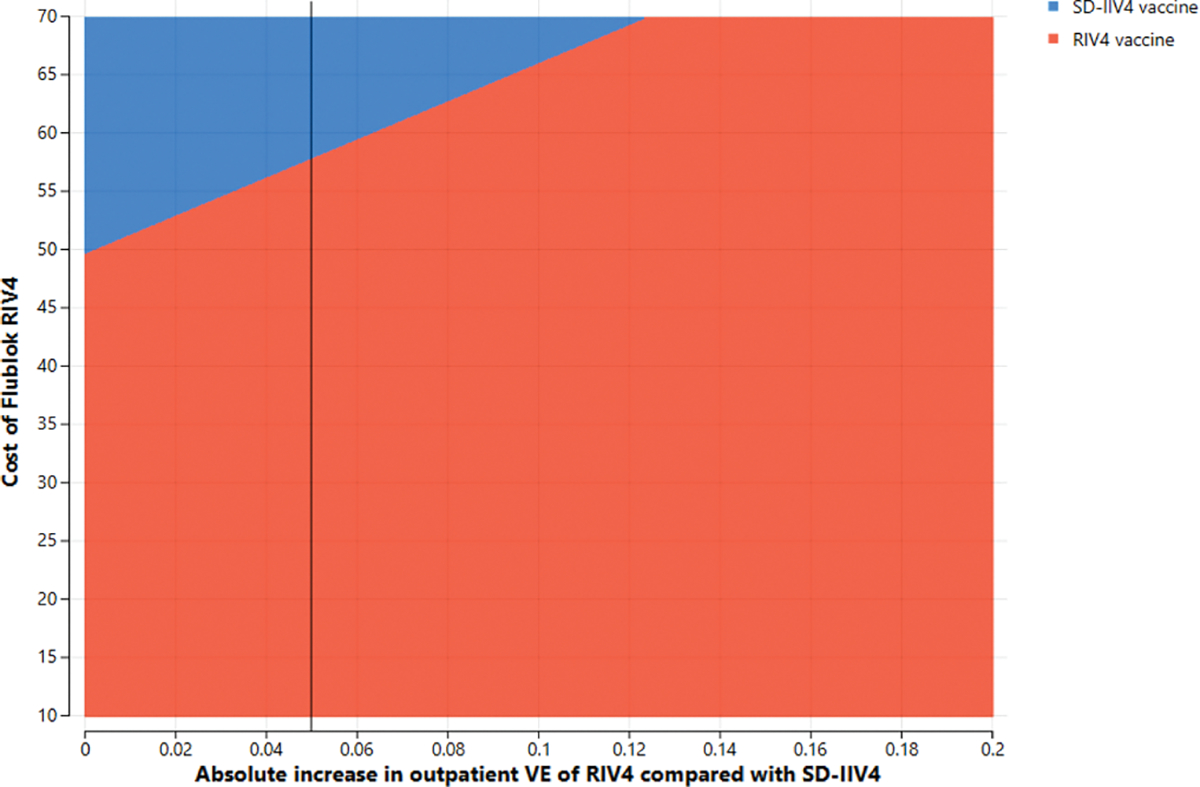
Two-way sensitivity analysis of absolute difference in *outpatien*t vaccine effectiveness between RIV4 and SD-IIV4 by cost of a dose of RIV4 in 18–64-year-olds at a $100,000/Qaly gained willingness-to-pay threshold.

**Fig. 4a.**
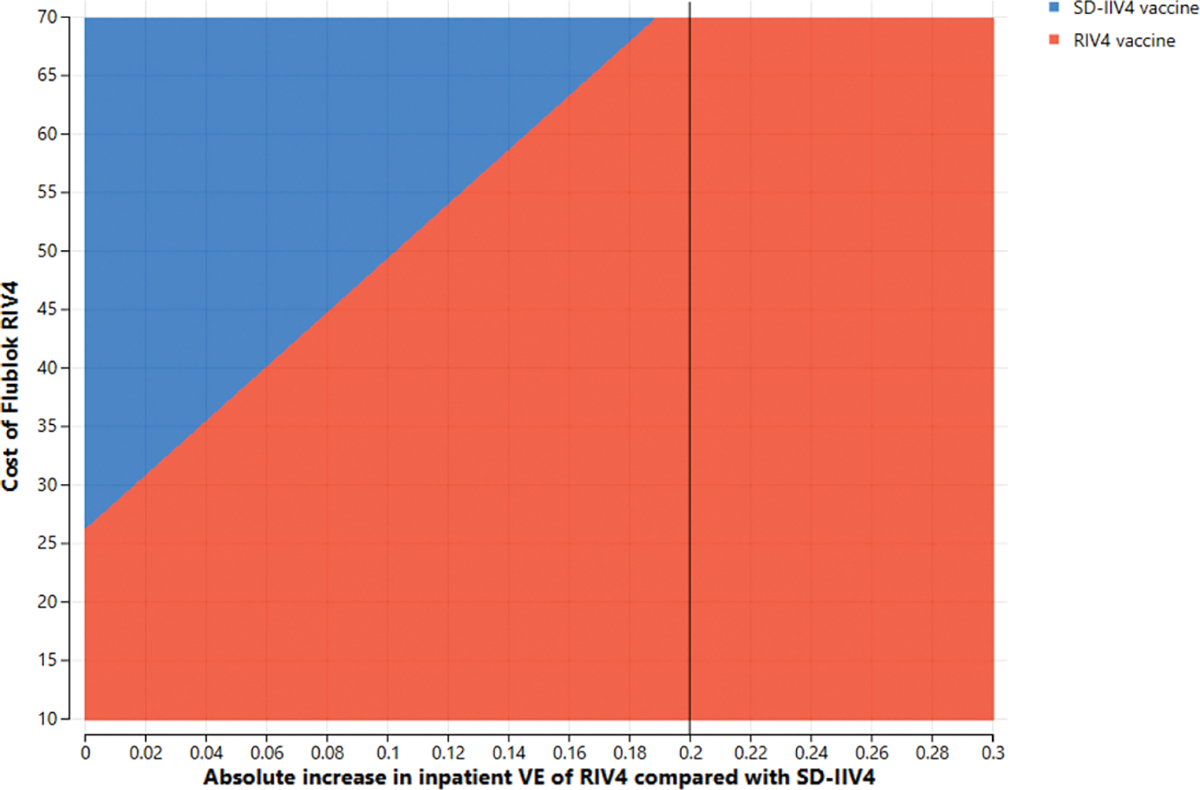
Two-way sensitivity analysis of absolute difference in *inpatient* vaccine effectiveness between RIV4 and SD-IIV4 by cost of a dose of RIV4 in 50–64-year-olds at a $100,000/QALY gained willingness-to-pay threshold.

**Fig. 4b.**
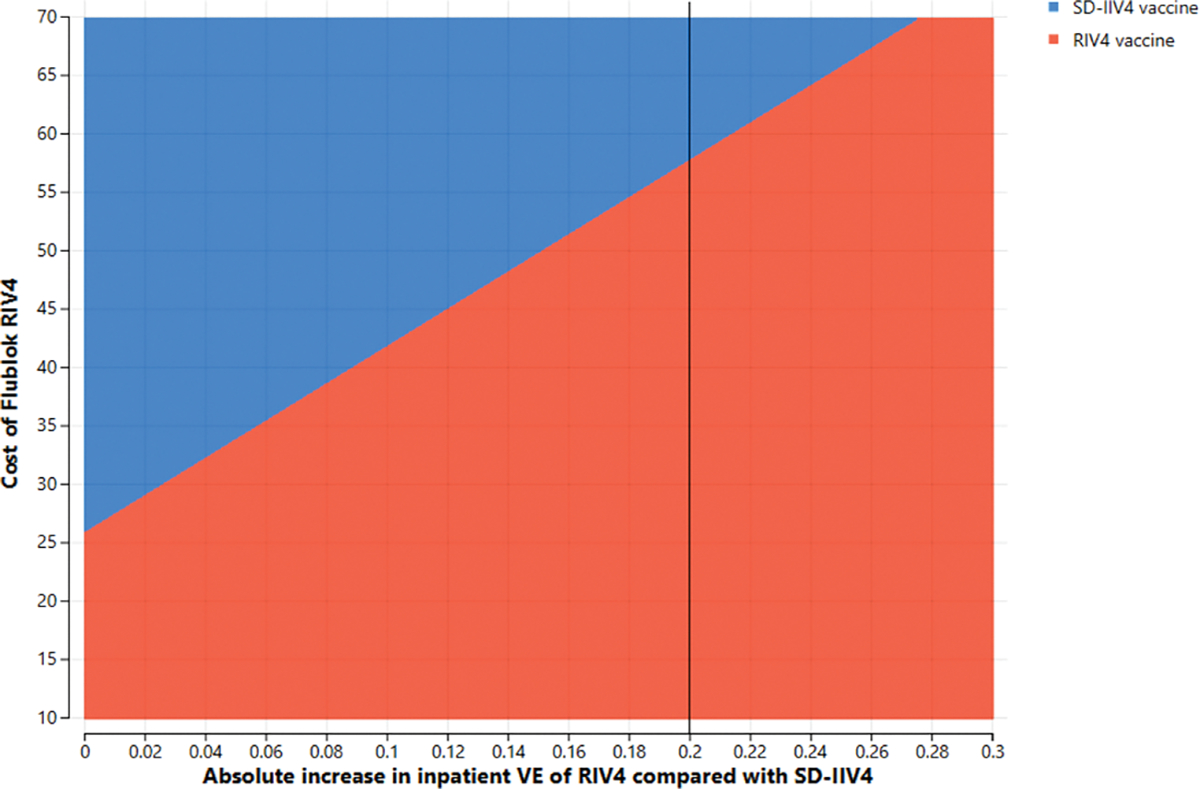
Two-way sensitivity analysis of absolute difference in *inpatient* vaccine effectiveness between RIV4 and SD-IIV4 by cost of a dose of RIV4 in 18–64-year-olds at a $100,000/QALY gained willingness-to-pay threshold.

**Table 1:** Variables used in Cost-Effectiveness Analyses.

	Description	Value	Low	High	Source

Constant					
	Inflation 2009–2020	1.21	0.60	1.80	[21]
	Inflation 2003–2020	1.41	0.70	2.11	[21]
Varying					
Costs & resource use, $					
Influenza illness					
Adverse event					
	Local	9.49	4.75	14.24	[22]
	Systemic	224	112	336	[8]
	Anaphylaxis	919	459	1378	[8]
	Guillain-Barre Syndrome	92,952	46,476	13,9428	[8]
Hospitalization					
	Societal cost of hospitalized influenza	211	102	306	[8]
	Not High Risk	27,211	13,606	40,817	[8]
	High Risk	35,622	17,811	53,434	[8]
Death					
	Not High Risk	79,830	39,916	119,748	[8]
	High Risk	86,649	43,325	129,976	[8]
Non-hospitalized					
	Self-treatment	4.83	2.41	7.24	[8]
	Not High Risk	509	255	764	[8]
	High Risk	802	401	1203	[8]
Vaccination					
	Vaccine administration	14.44	7.22	21.66	[23]
	Cost of Fluzone SD-IIV4 CPT: 90,688	17.84	8.92	26.75	[23]
	Cost of Flublok RIV4 CPT: 90,682	56.06	10.00	70.00	[23]
Durations (days, except as noted)					
Vaccination					
Adverse event					
	Local	0.1000	0	0.70	[24]
	Anaphylaxis	7	0	0.70	[24]
	Symptomatic influenza	10	7.50	12.50	[24]
Hospitalization duration					
	Hospitalization – uncomplicated	5	3.75	6.25	[24]
	Hospitalization – ICU	10	7.5	12.5	[24]
Influenza death (discounted life years lost					
	@ 3 %/year)	41.48	25.5	65.0	[25]
Probability					
Influenza illness		0.1394	0.06125	0.26	[20]
Case-hospitalization					
	Age 18–64 years	0.00748	0.005	0.01	[20]
	Age 50–64 years	0.0106	0.005	0.015	[20]
Proportion high risk, given hospitalization					
	Age 18–64 years	0.262	0.13	0.39	[7]
	Age 50–64 years	0.334	0.19	0.37	[7]
Case-fatality					
	Age 18–64 years	0.0004	0.0002	0.0005	[20]
	Age 50–64 years	0.0007	0.0005	0.0009	[20]
Proportion high risk, given fatality					
	Age 18–64 years	0.303	0.15	0.42	[7]
	Age 50–64 years	0.334	0.19	0.37	[7]
Outpatient visit, given illness					
	Age 18–64 years	0.39	0.20	0.50	[20]
	Age 50–64 years	0.42	0.30	0.50	[20]
Proportion high risk, given office visit					
	Age 18–64 years	0.326	0.19	0.56	[7]
	Age 50–64 years	0.501	0.33	0.67	[7]
Vaccination					
	Influenza vaccine uptake 2019–2020	0.4810	0.2405	0.7215	[26]
Adverse event					
	Anaphylaxis	0.00000025	0.00000017	0.00000051	[24]
	Guillain-Barre Syndrome	0.00000100	0.0000005	0.0000015	[24]
	Systemic	0.011	0.0055	0.0165	[24]
	Local	0.44	0.22	0.66	[24]
Effectiveness compared to no vaccination					
	Adjusted SD-IIV4 VE against hospitalization	0.27	0.08	0.42	[4]
	Adjusted SD-IIV4 VE against outpatient illness	0.35	0.185	0.555	[2]
	Adjusted RIV4 VE against hospitalization	0.47	0.35	0.56	[4]
	Adjusted RIV4 VE against outpatient illness	0.40	0.25	0.51	[2]
Utility					
Influenza illness					
	Hospitalized influenza	0.514	0.43	0.55	[27]
	Non-hospitalized influenza	0.659	0.55	0.77	[27]

**Table 2:** Primary outcomes of cost-effectiveness analysis.

	Strategy	Cost	Increased Cost	Effectiveness	Increased Effectiveness	ICER*

*Ages 18–64 years*						
*Base case*	SD-IIV4	$75.54	–	0.06038	–	–
	RIV4	$89.91	$14.37	0.06023	0.00015	$94,186
*Ages 50–64 years*						
*Base case*	SD-IIV4	$94.86	–	0.06087	–	–
	RIV4	$107.53	$12.67	0.06066	0.00021	$61,329

* ICER = Incremental Cost-effectiveness Ratio.

**Table 3:** One-way sensitivity analysis. Parameters whose variation changed RIV4 favorability at a $100,000/ quality adjusted life year threshold*.

Parameter	Baseline Value	Value at Threshold	
	
		*18–64-year-olds*	*50–64-year-olds*

RIV4 VE hospitalization	47 %	45.9 %	39.9 %
SD-IIV4 VE hospitalization	27 %	27.5 %	33.8 %
SD-IIV4 VE outpatient illness	35 %	35.8 %	44.3 %
Influenza attack rate (18–64 years)	13.9 %	13.8 %	10.2 %
RIV4 cost	$56.06	$57.90	–^†^
SD-IIV4 cost	$17.84	$15.99	–^†^
Probability of death given infection	0.038 %	0.035 %	–^†^
RIV4 VE outpatient illness	40 %	38.9 %	–^†^
Probability of hospitalization given infection	0.75 %	0.52 %	–^†^
Outpatient influenza utility value	0.659	0.758	–^†^

SD-IIV4 = quadrivalent standard dose influenza vaccine.

RIV4 = quadrivalent recombinant influenza vaccine.

* Listed from greatest to least change in the incremental cost-effectiveness ratio with parameter variation in 50–64-year-olds.

† No threshold value found within Table 1 range.

**Table 4:** Projected annual public health effects of influenza vaccination strategies in U.S. adults 18–64 years old and 50–64 years old.

Number of influenza events assuming 40 % vaccine uptake				

	Deaths	Hospitalizations	Outpatient visits	Total cases

*18–64-year-olds (N = 199,841,852)*				
SD-IIV4	9,211	181,316	9,035,546	23,168,068
RIV4	8,193	161,270	8,774,253	22,498,084
Difference	1,018	20,046	261,293	669,984
*50–64-year-olds (N = 62,788,435)*				
SD-IIV4	5,407	80,730	3,130,051	7,279,189
RIV4	4,810	71,804	3,039,535	7,068,687
Difference	597	8,926	90,516	210,502

SD-IIV4 = quadrivalent standard dose influenza vaccine.

RIV4 = quadrivalent recombinant influenza vaccine.

## Data Availability

No data was used for the research described in the article.
